# Genetic analysis and QTL mapping of aroma volatile compounds in the apple progeny ‘Fuji’ × ‘Cripps Pink’

**DOI:** 10.3389/fpls.2023.1048846

**Published:** 2023-03-20

**Authors:** Shunbo Yang, Jing Yu, Huijuan Yang, Zhengyang Zhao

**Affiliations:** ^1^ College of Horticulture, Northwest A & F University, Yangling, China; ^2^ Shaanxi Research Center of Apple Engineering and Technology, Yangling, China

**Keywords:** apple, aroma, QTL mapping, candidate gene, flavor

## Abstract

Aroma is an essential trait for apple fruit quality, but the understanding of biochemical mechanisms underlying aroma formation is still limited. To better characterize and assess the genetic potential for improving aroma quality for breeding, many efforts have been paid to map quantitative trait loci (QTLs) using a saturated molecular linkage map. In the present study, aroma profiles in ripe fruit of F_1_ population between ‘Fuji’ and ‘Cripps Pink’ were evaluated by gas chromatography-mass spectrometry (GC-MS) over 2019 and 2020 years, and the genetics of volatile compounds were dissected. In total, 38 volatile compounds were identified in ‘Fuji’ × ‘Cripps Pink’ population, including 23 esters, 3 alcohols, 7 aldehydes and 5 others. With the combination of aroma phenotypic data and constructed genetic linkage map, 87 QTLs were detected for 15 volatile compounds on 14 linkage groups (LGs). Among them, a set of QTLs associated with ester production identified and confirmed on LG 6. A candidate gene *MdAAT6* in the QTL mapping interval was detected. Over-expression of *MdAAT6* in tomato and apple fruits showed significantly higher esters accumulation compared to the control, indicating it was critical for the ester production. Our results give light on the mode of inheritance of the apple volatilome and provide new insights for apple flavor improvement in the future.

## Introduction

Apples (*Malus* × *domestica* Borkh.) are widely cultivated all over the world because of the highly flavored fruits with unique flavor characteristics (Elss et al., 2006; Yang et al., 2021a). Flavor is the key driver of consumers’ appreciation for fruits and generally determined by sugars, organic acids and aroma (Klee et al., 2010). Among these, aroma is a complex mixture of many volatile compounds, which directly contribute to the perceived odour and sensory quality of fruits (Espino-Diaz et al., 2016; Yang et al., 2021b). In apples, more than 350 volatile compounds represented broadly as esters, alcohols, aldehydes, ketons and sesquiterpenes have been detected and identified (Dixon and Hewett, 2000; Song and Forney, 2008). However, only a subset of 20–30 compounds appear to dominate the typical apple aroma (Aprea et al., 2012). Character impact odorants reported from apple fruits include hexanol, (*E*)-2-hexenal, α-farnesene, estragole, esters like hexyl acetate, hexyl hexanoate and 2-methylbutyl acetate (Mehinagic et al., 2006; Yan et al., 2020; Liu et al., 2021). The apple aroma volatile compounds are produced by four main biochemical pathways: the fatty acid pathway contributing to straight chain esters, the isoleucine biosynthesis pathway contributing to branched chain esters, the α-farnesene synthesis pathway and the phenylpropanoid pathway (Schaffer et al., 2007). In contrast to the knowledge about the analytical and biochemical backgrounds of aroma volatiles, little is known about the genetic bases and inheritance patterns in apple fruit.

Aroma formation in apple fruit is a dynamic process involving variations in the composition and concentration of volatile profiles depending on maturity stage, postharvest factors and genotype. Headspace solid–phase microextraction (HS-SPME) combined with gas chromatography–mass spectrometry (GC-MS), as a simple, rapid, comprehensive and high sensitivity method for the volatile compounds assessment, has been widely used for the extraction of volatile profiles in apple fruit (Ban et al., 2010; Zhu et al., 2020; Yang et al., 2021b). With the advances in qualitative and quantitative methods for measuring volatile compounds, improvement of fruit aroma has been considered a priority in many breeding programs (Klee, 2010; Cappellin et al., 2015a). Apple is a self-incompatible and highly heterozygous species, cross breeding may result in a amount of diverse progeny (Rowan et al., 2009). Therefore, considering the long juvenility period of apple, the traditional process of breeding a new apple variety solely based on phenotypic selection is time-consuming, expensive and somewhat inefficient. Marker-assisted selection (MAS) can greatly reduce breeding costs and improve breeding efficiency (Xu et al., 2012). Based on the construction of genetic linkage maps, mapping of quantitative trait loci (QTLs) controlling fruit aroma volatile levels and subsequent identification of linked molecular markers or key genes is a crucial goal for future marker-assisted selection in apple breeding programs.

To date, several QTL mapping researches have been carried out in cultivated apples in order to detect genomic areas involved in the inheritance of aroma volatiles. Taking advantage of the saturated genetic linkage map, Zini et al. (2005) reported the first preliminary volatile QTL detection results using the ‘Fiesta’ × ‘Discovery’ apple progeny. Furthermore, Dunemann et al. (2009a) investigated the aroma compounds with headspace solid-phase microextraction gas chromatography in the apple progeny ‘Discovery’ × ‘Prima’ and the QTL mapping results showed that the QTLs were mainly clustered on linkage groups LG 2, 3 and 9. In another attempt, an AAT (Alcohol acyl transferase) candidate gene was genetically mapped on LG 2 and found to be associated with a QTL cluster highly impacting apple aroma of four key ester compounds pentyl acetate, butyl acetate, hexyl acetate, and 2-methyl-butyl acetate (Dunemann et al., 2009b). In addition, Costa et al. (2013) confirmed and validated a set of QTLs associated to volatile organic compounds in apple under three different environments in Switzerland, and the presence of important QTLs about esters and hormone ethylene were found to be located in the linkage group 2 and 15, respectively. Souleyre et al. (2014) identified 46 QTLs on 15 linkage groups (LGs) for the production of esters and alcohols in ‘Royal Gala’ × ‘Granny Smith’ population and the major QTL for 35 volatiles was positioned on LG2 and co-located with AAT1, which catalyzed the synthesis of esters contributing to the ‘ripe apple’ flavour. However, since the release of ‘Golden Delicious’ apple new reference genome (Daccord et al., 2017), to our knowledge, no further investigation about the genetic dissection of apple aroma traits or identification for the key genes involved in the aroma synthesis by the QTL-based approach has been reported.

In this study, an F_1_ population of ‘Fuji’ × ‘Cripps Pink’ was used to dissect the genetics underlying aroma volatile compounds with HS–SPME/GC–MS and locate the QTL regions to further verify the candidate genes that affect the volatile accumulation in apple fruit. These findings lay a foundation for analyses of the genetic mechanisms underlying aroma volatiles and the breeding of apple varieties with better flavor in the future.

## Materials and methods

### Plant materials

An F_1_ segregating population with 300 individuals was developed from the cross of ‘Fuji’ × ‘Cripps Pink’. The 300 lines of this population were grafted onto dwarf rootstock M26 and planted at a density of 1.5 m × 4 m at the Baishui Apple Experimental Station of Northwest A & F University, Shaanxi Province, China. Orchard management procedures such as irrigation, fertilisation and pruning, were similar for all apple trees. The apple fruits from 246 and 223 individuals were harvested in two growing years (2019 and 2020). Three biological replicates with six fruits for each individual were collected at the ripe stage. Slices of flesh tissue were separated, immediately frozen in liquid nitrogen and stored at -80°C until use.

### Determination of volatiles

Fruit volatile compounds were analyzed according to the method reported by Yang et al. (2021a). For extraction of volatile compounds, the apple flesh tissue (5 g) was ground to a powder in liquid nitrogen, then transferred into a 50 ml headspace vial containing a magnetic stirring rotor and 1 g NaCl spiked with 10 μL (0.4 mg/mL) 3-nonanone (internal standard). After the headspace vial equilibrated at 50°C for 10 min on a metal heating agitation platform, a 2.0 cm SPME fiber (50/30 µm, DVB/CAR/PDMS; Supelco, Bellefonte, PA, USA) was inserted into the headspace for 30 min at 50°C with agitation at 200 rpm. After extraction, the fiber was desorbed in the GC injection port at 250°C for 2.5 min.

A Thermo Trace GC Ultra gas chromatograph (Thermo Fisher Scientific, New York, NY, USA) equipped with an HP-INNOWax capillary column (60 m × 0.25 mm × 0.25 µm) was used to analyze the volatile compounds in samples. The oven programming conditions were: 40°C kept for 3 min, ramped at 5°C/min to 150°C, then increased at 10°C/min to 220°C and held for 5 min. The carrier gas was helium at a flow rate of 1.0 mL/min with the ion source and transfer line temperatures were both set at 240°C. Mass spectra were monitored from 35 to 450 m/z with ionizing electron energy of 70 eV. Volatile compounds were identified by comparison with retention times (RT), retention indices (RI) and the mass spectra of the NIST 14 library (NIST/EPA/NIH). Quantification of volatile compounds was performed using the peak area of the internal standard as a reference based on the total ion chromatogram (TIC).

### Linkage map construction, QTL mapping and candidate gene annotation

An F_1_ segregating population with 300 individuals from the cross of ‘Fuji’ × ‘Cripps Pink’ was utilized to construct a high-density genetic linkage map by whole genome resequencing (WGS) in the previous study (Liu, 2022). QTL mapping was carried out with Map QTL software (version 5.0) using composite interval mapping (CIM) methods. The logarithm of odds (LOD) threshold for declaring effective QTLs was determined using a permutation test (1000 replications) with a significance level of *P* < 0.05 and the limit of detection LOD threshold was set at 3.0. Designations for QTLs were started with q, followed by the abbreviation of volatiles name, the years and the QTL order along the chromosome. The functional annotations of candidate genes in the QTL regions were obtained on the basis of the *Malus* genome GDDH13 version 1.1 (https://iris.angers.inra.fr/gddh13).

### RNA extraction and qRT-PCR analysis

In order to know more about the expression patterns of candidate gene, total RNA of different development stages (60, 90, 120, 150, 180 days after full bloom) and tissues (leaf, root, stem, peel, pulp) in ‘Fuji’ apple was extracted with a Plant RNA Purification Kit (Tiangen, China) following the manufacturer’s instructions. cDNA was obtained *via* reverse transcription using the PrimeScript™ RT Reagent Kit (Perfect Real Time) (Takara, Japan). The qRT-PCR (quantitative real-time reverse transcription PCR) experiment was performed using an iQ5 Multicolor Real-Time PCR Detection System (Bio-Rad, Hercules, CA, USA). *MdActin* was used for target gene normalization in accordance with 2^-ΔΔCt^ method to calculate the relative expression level. Three replicates per each sample were performed. The gene-specific primers used for qRT-PCR are listed in **Table S1**.

### Subcellular localization

The coding sequence of *MdAAT6* without the stop codon was inserted into a pCAMBIA2300-GFP vector under the control of the CaMV 35S promoter, and the primers used are listed in **Table S1**. The fusion plasmids and control vector (pCAMBIA2300-GFP) were separately introduced into *Nicotiana benthamiana* leaf by *Agrobacterium*-mediated infiltration according to the method by Sainsbury et al. (2009). After 3 days of infiltration, fluorescence was visualized with the confocal laser-scanning microscope (LSM 710, Carl Zeiss, Oberkochen, Germany) at excitation wavelength of 488 nm.

### Transient expression in apple fruits

*Agrobacterium*-mediated transient transformation in apple fruits was carried out with minor modifications from the procedure of Chen et al. (2021). The coding sequence of candidate gene was amplified and cloned into the plant expression vectors pCAMBIA2300 and TRV2 for over-expression and silent expression in apple fruits. The constructs and vector-only controls were transformed by heat shock into *Agrobacterium tumefaciens* strain GV3101. The agrobacteria cell suspension was evenly injected into the apple fruits with a syringe. The injected apple fruits were placed in paper bags and kept in the darkness for 12 h and then transferred to a light growth chamber without the bags at 26°C for 5 days. Fruits were injected with the empty vectors, used as the negative controls. After 5 days, the infiltrated fruits were collected for aroma volatile compounds determination. Three biological replicates with 6 fruits each were used for analysis.

### Stable over-expression in tomato

To generate transgenic tomato plants, the coding region of candidate gene from *Malus domestica* cv. ‘Fuji’ was cloned into pCAMBIA2300 vector. *Agrobacterium*-mediated tomato (*Solanum lycopersicum* cv. Micro-Tom) transformation was performed as described by Hao et al. (2015). The T3 generation of the homozygous transgenic lines was screened by Kan resistance and PCR analysis. The fruits of tomato were harvested at the full maturity stage for the further GC–MS analysis.

### Statistical analysis

All data were obtained from three replications. Statistical analyses were performed using the IBM SPSS Statistics 21 (IBM, Armonk, NY, USA) and Excel 2019 software. The data were analyzed using Student t-test or one-way analysis of variance (ANOVA), and significant differences between groups were assessed by the Tukey test (*P* < 0.05).

## Results

### Volatile profiling and variation in F1 population of ’Fuji‘ × ’Cripps Pink‘

In order to reveal the genetic characteristics of apple aroma, the composition and content of volatiles were evaluated by GC-MS over two successive years in ripe fruit of F_1_ population between ‘Fuji’ and ‘Cripps Pink’. In total, 38 volatile compounds were identified in female parent (‘Fuji’) and male parent (‘Cripps Pink’), including 23 esters, 3 alcohols, 7 aldehydes and 5 others (**Table 1**). Butyl acetate, 2-methylbutyl acetate, butyl 2-methylbutanoate, hexyl acetate, hexyl 2-methylbutyrate, 2-methyl-1-butanol, 1-hexanol, hexanal and (*E*)-2-hexenal were the most abundant compounds (average content > 10 µg/kg FW in 2019 and 2020) in ‘Fuji’ apple. While in ‘Cripps Pink’ apple, 2-methylbutyl acetate, hexyl acetate, hexyl butanoate, hexyl 2-methylbutyrate, (*E*)-2-hexenal, estragole and α-farnesene showed the highest contents (more than 10 µg/kg FW in two years). Moreover, esters were the dominant aromatic compounds in both parents in two years, accounting for 51.14% (‘Fuji’, 2019), 66.28% (‘Fuji’, 2020), 53.56% (‘Cripps Pink’, 2019), 67.45%, (‘Cripps Pink’, 2020) of the total volatile content, respectively (**Figure S1**). In addition, ‘Fuji’ had a higher proportion of aldehyde compounds, which contributed to 34.98% and 17.18% in 2019 and 2020, while ‘Cripps Pink’ had a lower aldehyde proportion of 15.93% (2019) and 4.78% (2020), respectively. The content and percentage of alcohols in ‘Cripps Pink’ apple (18.69 μg/kg FW, 4.04%; 23.94 μg/kg FW, 5.03%) were all lower than that in ‘Fuji’ apple (36.67 μg/kg FW, 11.78%; 40.32 μg/kg FW, 11.21%) during two years.

**Table 1:** Composition, content (μg/kg FW) and percentage (%) of volatile compounds in parent fruits.

Types	Volatile compounds	CAS No^a^	RI^b^	Fuji (Female parent)		Cripps Pink (Male parent)	
				2019	2020	2019	2020
*Esters*	Propyl acetate	109-60-4	982	1.17 ± 0.21 (0.38%)	1.00 ± 0.18 (0.28%)	1.52 ± 0.18 (0.33%)	1.42 ± 0.23 (0.30%)
	Ethyl butanoate	105-54-4	1045	1.19 ± 0.15 (0.38%)	0.41 ± 0.05 (0.11%)	1.11 ± 0.24 (0.24%)	0.23 ± 0.06 (0.05%)
	Propyl propionate	106-36-5	1050	0.82 ± 0.10 (0.26%)	1.20 ± 0.16 (0.33%)	1.41 ± 0.20 (0.30%)	2.56 ± 0.18 (0.54%)
	Ethyl 2-methylbutanoate	7452-79-1	1062	0.73 ± 0.07 (0.23%)	0.35 ± 0.07 (0.10%)	nd	nd
	Butyl acetate	123-86-4	1074	15.72 ± 2.39 (5.05%)	19.37 ± 1.38 (5.38%)	8.87 ± 0.94 (1.92%)	6.38 ± 0.62 (1.34%)
	2-Methylbutyl acetate	624-41-9	1126	54.98 ± 5.67 (17.67%)	71.60 ± 8.02 (19.90%)	17.98 ± 1.21 (3.88%)	15.37 ± 1.82 (3.23%)
	Isobutyl butanoate	539-90-2	1155	0.15 ± 0.02 (0.05%)	3.35 ± 0.36 (0.93%)	nd	nd
	Amyl acetate	628-63-7	1178	2.96 ± 0.34 (0.95%)	4.57 ± 0.51 (1.27%)	2.02 ± 0.30 (0.44%)	1.77 ± 0.19 (0.37%)
	Butyl butanoate	109-21-7	1240	2.59 ± 0.25 (0.83%)	9.26 ± 1.52 (2.57%)	3.42 ± 1.02 (0.74%)	12.12 ± 5.78 (2.55%)
	Butyl 2-methylbutanoate	15706-73-7	1243	13.26 ± 1.25 (4.26%)	17.14 ± 2.41 (4.76%)	9.15 ± 1.11 (1.98%)	12.70 ± 1.52 (2.67%)
	3-Methylbutyl butanoate	106-27-4	1270	0.30 ± 0.05 (0.10%)	0.81 ± 0.17 (0.23%)	nd	nd
	Hexyl acetate	142-92-7	1274	38.20 ± 4.05 (12.28%)	64.10 ± 6.22 (17.82%)	148.02 ± 20.47 (31.98%)	171.95 ± 22.38 (36.13%)
	2-Methylbutyl 2-methylbutyrate	2445-78-5	1286	1.37 ± 0.20 (0.44%)	1.37 ± 0.14 (0.38%)	nd	nd
	Pentyl butanoate	540-18-1	1321	0.10 ± 0.03 (0.03%)	0.64 ± 0.05 (0.18%)	nd	nd
	Propyl hexanoate	626-77-7	1324	2.24 ± 0.32 (0.72%)	2.57 ± 0.28 (0.71%)	2.82 ± 0.36 (0.61%)	2.98 ± 0.52 (0.63%)
	Amyl 2-methylbutyrate	68039-26-9	1330	0.75 ± 0.08 (0.24%)	1.15 ± 0.20 (0.32%)	nd	nd
	Hexyl propanoate	2445-76-3	1347	0.73 ± 0.10 (0.23%)	1.27 ± 0.16 (0.35%)	1.27 ± 0.33 (0.27%)	1.36 ± 0.28 (0.29%)
	Hexyl isobutyrate	2349-07-7	1350	nd	nd	0.21 ± 0.02 (0.05%)	0.19 ± 0.05 (0.04%)
	Heptyl formate	112-23-2	1357	nd	nd	0.61 ± 0.08 (0.13%)	0.23 ± 0.05 (0.05%)
	Butyl hexanoate	626-82-4	1410	0.87 ± 0.10 (0.28%)	2.56 ± 0.54 (0.71%)	1.20 ± 0.22 (0.26%)	2.20 ± 0.31 (0.46%)
	Hexyl butanoate	2639-63-6	1423	5.16 ± 0.31 (1.67%)	7.35 ± 2.52 (2.04%)	21.88 ± 3.69 (4.73%)	53.90 ± 10.25 (11.33%)
	Hexyl 2-methylbutyrate	10032-15-2	1438	15.17 ± 2.65 (4.87%)	25.56 ± 3.66 (7.10%)	24.35 ± 2.58 (5.26%)	33.31 ± 4.20 (7.00%)
	Hexyl hexanoate	6378-65-0	1593	0.67 ± 0.08 (0.22%)	2.86 ± 0.25 (0.79%)	2.06 ± 0.30 (0.45%)	2.36 ± 0.42 (0.50%)
*Alcohols*	1-Butanol	71-36-3	1156	3.21 ± 0.21 (1.03%)	5.81 ± 0.14 (1.61%)	7.91 ± 0.51 (1.71%)	15.36 ± 0.82 (3.23%)
	2-Methyl-1-butanol	137-32-6	1210	11.31 ± 0.82 (3.63%)	11.71 ± 2.33 (3.25%)	4.26 ± 0.27 (0.92%)	0.85 ± 0.02 (0.18%)
	1-Hexanol	111-27-3	1361	22.15 ± 1.25 (7.12%)	22.80 ± 3.48 (6.34%)	6.52 ± 0.40 (1.40%)	7.73 ± 0.62 (1.62%)
*Aldehydes*	Hexanal	66-25-1	1090	33.19 ± 1.86 (10.67%)	9.44 ± 0.75 (2.62%)	15.36 ± 1.62 (3.32%)	1.98 ± 0.53 (0.41%)
	(*E*)-2-Hexenal	6728-26-3	1240	71.32 ± 6.52 (22.92%)	47.51 ± 4.24 (13.20%)	55.21 ± 4.81 (11.93%)	16.17 ± 1.22 (3.40%)
	Octanal	124-13-0	1298	nd	nd	0.32 ± 0.02 (0.07%)	0.45 ± 0.03 (0.09%)
	Nonanal	124-19-6	1401	0.60 ± 0.05 (0.19%)	0.47 ± 0.03 (0.13%)	0.87 ± 0.08 (0.19%)	1.62 ± 0.27 (0.34%)
	(*E*)-2-Octenal	2548-87-0	1443	1.25 ± 0.14 (0.40%)	1.33 ± 0.10 (0.37%)	1.32 ± 0.06 (0.28%)	0.85 ± 0.05 (0.18%)
	Decanal	112-31-2	1480	1.63 ± 0.22 (0.52%)	1.73 ± 0.17 (0.49%)	0.21 ± 0.02 (0.05%)	0.69 ± 0.11 (0.14%)
	(*Z*)-2-Nonenal	60784-31-8	1531	0.85 ± 0.10 (0.27%)	1.34 ± 0.24 (0.38%)	0.43 ± 0.03 (0.09%)	0.99 ± 0.15 (0.21%)
*Others*	Pentylcyclopropane	2511-91-3	975	0.21± 0.04 (0.07%)	0.66 ± 0.08 (0.18%)	3.51 ± 0.29 (0.76%)	1.05 ± 0.07 (0.22%)
	1-Octen-3-one	4312-99-6	1305	0.24 ± 0.02 (0.08%)	0.57 ± 0.07 (0.17%)	0.21 ± 0.03 (0.05%)	0.52 ± 0.04 (0.11%)
	Estragole	140-67-0	1687	0.63 ± 0.29 (0.20%)	7.65 ± 0.85 (2.13%)	77.48 ± 10.52 (16.73%)	50.89 ± 8.49 (10.69%)
	α-Farnesene	502-61-4	1725	5.21 ± 0.38 (1.68%)	8.40 ± 1.07 (2.33%)	37.62 ± 3.54 (8.13%)	51.49 ± 6.33 (10.82%)
	(*Z*)-Anethole	25679-28-1	1780	0.26 ± 0.07 (0.08%)	1.89 ± 0.25 (0.54%)	3.70 ± 0.26 (0.80%)	4.25 ± 0.51 (0.88%)

^a^CAS number; ^b^Retention Index. Datas are the mean value ± standard deviation of three biological replicates; FW, fresh weight; nd, not detected.

**Figure 1:**
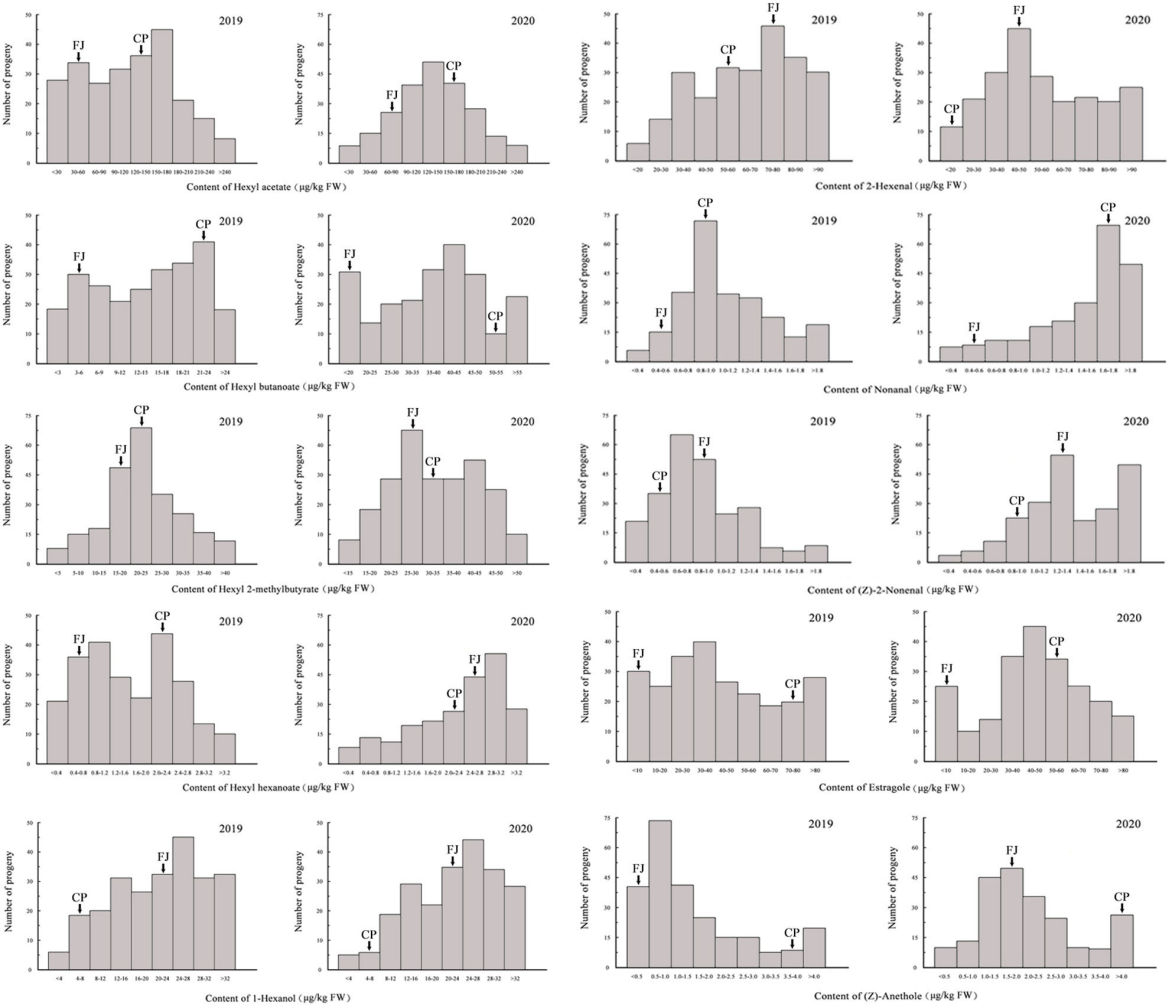
Frequency distributions of 10 volatile compounds among F_1_ progeny fruits of ‘Fuji’ (FJ) × ‘Cripps Pink’ (CP) population in 2019 and 2020. Arrows indicated the contents for two parents.

In the ‘Fuji’ × ‘Cripps Pink’ population, the volatiles present in both parents all could be detected in the offspring. A total of 15 volatile compounds were unsegregated in F_1_ progeny fruits and their parents (**Table 2**), distributed across distinct chemical classes: esters (4), alcohols (2), aldehydes (5) and others (4). Among them, hexyl acetate, (*E*)-2-hexenal and α-farnesene were the most abundant volatiles (average content > 40 µg/kg FW over two years) in F_1_ progeny fruits and the mean contents of them were 95.80, 78.81, 43.73 μg/kg FW in 2019 and 128.94, 72.63, 90.30 μg/kg FW in 2020, respectively. Moreover, the relative contents of α-farnesene, estragole, hexyl 2-methylbutyrate and hexyl butanoate were widely distributed among F_1_ progeny fruits, ranging from 0.20 to 866.93 µg/kg FW, 0.23 to 684.34 µg/kg FW, 0.21 to 431.52 µg/kg FW, 0.11 to 370.66 µg/kg FW, respectively. Additionally, as shown in **Figure 1**, transgressive segregation occurred in both directions and was observed for 10 volatile compounds (hexyl acetate, hexyl butanoate, hexyl 2-methylbutyrate, hexyl hexanoate, 1-hexanol, (*E*)-2-hexenal, nonanal, (*Z*)-2-nonenal, estragole and (*Z*)-anethole), with a relative range of variation between 42.96% and 187.16% along the two years. These unsegregated compounds showed continuous variation, typical of polygenic inheritance, although their distributions were generally skewed toward high values. Also, a continuous distribution and considerable transgressive segregation observed in the F_1_ population demonstrated that both parents contributed alleles and these volatiles were confirmed to meet the requirements for further QTL mapping.

**Table 2:** Descriptive statistics for 15 unsegregated volatile compounds in F_1_ progeny fruits of ‘Fuji’ × ‘Cripps Pink’ population.

Types	Volatile compounds	Year	Mid-parent value^a^	F_1_ progeny				
				Min^b^	Max^c^	Mean^d^	SD^e^	CV (%)^f^
*Esters*	Hexyl acetate	2019	93.11	0.18	690.81	95.80	98.97	103.31
		2020	118.03	0.50	715.48	128.94	130.76	101.41
	Hexyl butanoate	2019	13.52	0.11	253.80	15.69	29.37	187.16
		2020	30.63	0.23	370.66	40.58	55.29	136.26
	Hexyl 2-methylbutyrate	2019	19.76	0.21	226.08	25.84	32.66	126.41
		2020	29.44	0.70	431.52	49.76	48.98	98.44
	Hexyl hexanoate	2019	1.37	0.25	86.62	2.29	2.40	104.95
		2020	2.61	0.21	104.03	7.36	8.31	112.93
*Alcohols*	2-Methyl-1-butanol	2019	7.79	0.41	33.71	6.24	5.36	85.97
		2020	6.28	0.42	87.10	10.52	9.04	85.91
	1-Hexanol	2019	14.34	1.04	101.34	29.41	27.41	93.19
		2020	15.27	2.08	313.31	45.47	50.95	112.05
*Aldehydes*	Hexanal	2019	24.28	0.73	59.65	42.63	40.65	95.36
		2020	5.71	1.82	108.13	20.59	18.25	88.65
	(*E*)-2-Hexenal	2019	63.27	1.16	167.75	78.81	64.66	82.04
		2020	31.84	1.70	174.43	72.63	58.61	80.69
	Nonanal	2019	0.74	0.27	3.20	1.16	0.82	70.38
		2020	1.05	0.35	4.87	1.70	1.31	76.95
	(*E*)-2-Octenal	2019	1.29	0.18	2.44	1.61	0.83	51.74
		2020	1.09	0.56	4.47	1.60	0.73	45.62
	(*Z*)-2-Nonenal	2019	0.64	0.19	1.89	0.91	0.39	42.96
		2020	1.17	0.32	4.86	2.09	0.99	47.44
*Others*	Pentylcyclopropane	2019	1.86	0.22	11.18	0.84	0.87	103.31
		2020	0.86	0.19	17.34	2.87	2.91	101.41
	Estragole	2019	39.06	0.23	684.34	31.28	58.54	187.16
		2020	29.27	0.25	649.42	45.98	62.65	136.26
	α-Farnesene	2019	21.42	0.20	543.38	43.73	55.28	126.41
		2020	29.95	0.49	866.93	90.30	88.89	98.44
	(*Z*)-Anethole	2019	1.98	0.21	21.54	1.65	1.73	104.95
		2020	3.07	0.16	19.65	2.10	2.37	112.93

^a^Average content (μg/kg FW) in parents. ^b^Lowest content (μg/kg FW) in F1 progeny fruits. ^c^Highest content (μg/kg FW) in F1 progeny fruits.

^d^Average content (μg/kg FW) in F1 progeny fruits. ^e^Standard deviation. ^f^Coefficient of variation.

During the two years, there were 14 volatile compounds (propyl acetate, ethyl butanoate, propyl propionate, butyl acetate, 2-methylbutyl acetate, amyl acetate, butyl butanoate, butyl 2-methylbutanoate, propyl hexanoate, hexyl propanoate, butyl hexanoate, 1-butanol, decanal, 1-octen-3-one) present in both parents, but separation occurred in the F_1_ progeny fruits of ‘Fuji’ × ‘Cripps Pink’ population (**Table S2**). Thereinto, the segregation ratio (1:1) of butyl hexanoate was confirmed by chi-square tests in two consecutive years of 2019 and 2020. Moreover, the butyl acetate segregation ratio matched a 1:3 ratio and the 2-methylbutyl acetate fit to the expected 1:15 segregation ratio as determined by a Chi-square test. These results indicated that those volatile compounds might be inherited qualitative traits, controlled by one gene.

### Correlation analysis for fruit aroma volatiles

**Figure 2:**
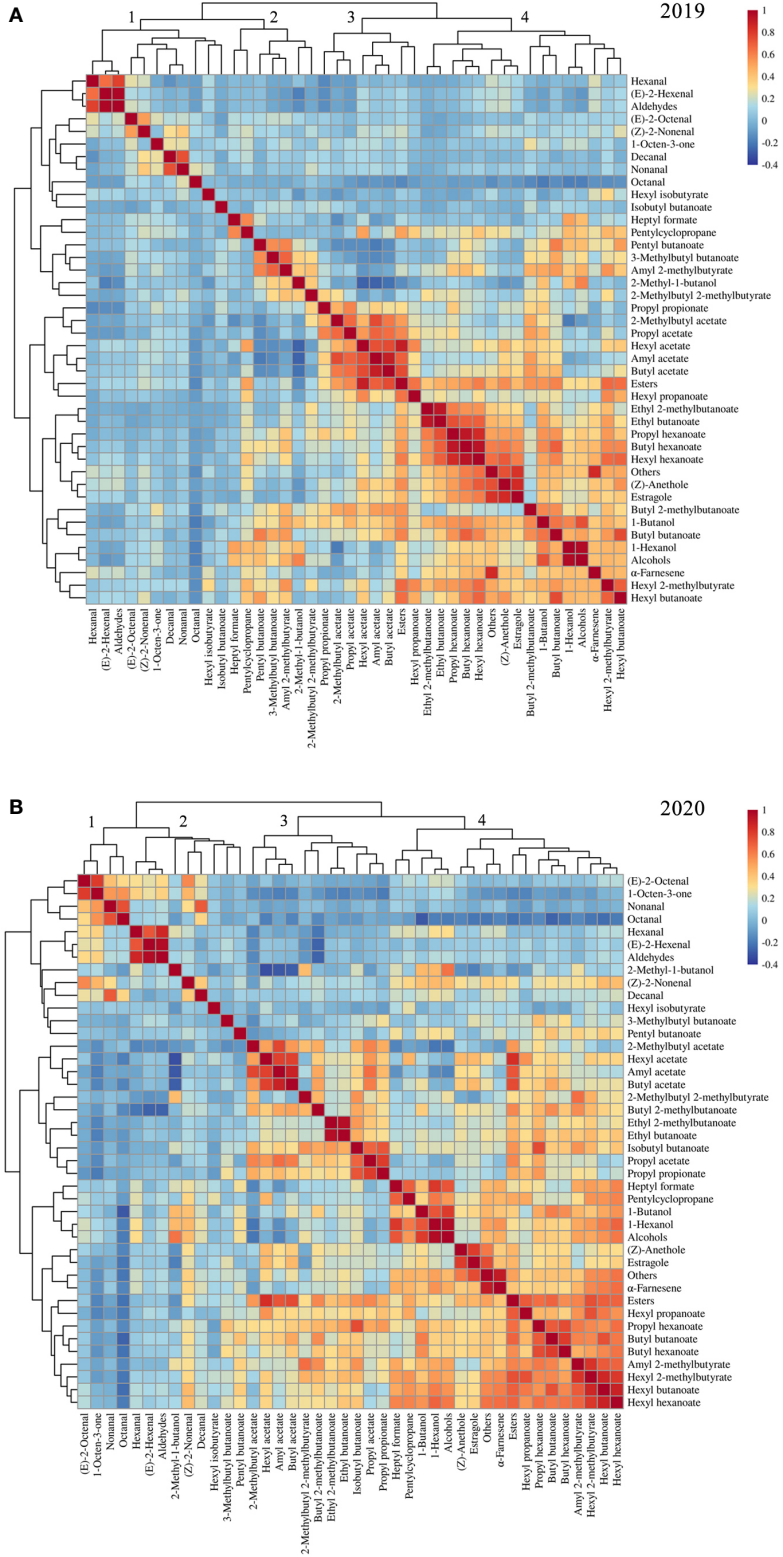
Heatmap and dendrogram showing the correlation matrix by the Pearson’s coefficient among volatile compounds in F_1_ progeny fruits of ‘Fuji’ × ‘Cripps Pink’ population for 2019 **(A)** and 2020 **(B)**.

Correlations among volatile compounds in the F_1_ progeny fruits of ‘Fuji’ × ‘Cripps Pink’ population were represented in heatmaps for 2019 (**Figure 2A**) and 2020 (**Figure 2B**). Volatiles belonging to the same biosynthetic pathway tended to be highly correlated. In two years, the ester compounds showed positive interactions with hexyl acetate, butyl acetate, hexyl 2-methylbutyrate, hexyl butanoate and hexyl hexanoate. The alcohol compounds were positively correlated with 1-butanol and 1-hexanol while negatively correlated with 2-methylbutyl acetate and octanal. Moreover, a strong positive relationship about aldehyde compounds between hexanal and (*E*)-2-hexenal was observed in the heatmap. Additionally, the content of aroma compounds belonging to other type in this study was highly positive correlation with estragole and α-farnesene.

The correlation analysis also revealed that volatiles in F_1_ population were clustered based on their compound classes and chemical properties. For hierarchical clustering, these volatile compounds roughly divided into four groups according to their aroma profiles across the two years (**Figures 2A, B**). Overall, cluster 1 was enriched with aldehyde compounds such as hexanal, (*E*)-2-hexenal and (*E*)-2-octenal. Cluster 2 showed enrichment of 2-methyl-1-butanol, pentyl butanoate and 3-methylbutyl butanoate. Cluster 3 mainly consists of some important esters (hexyl acetate, butyl acetate, amyl acetate and 2-methylbutyl acetate). Cluster 4 was grouped by the compounds with high levels in apple fruit, for instance, α-farnesene, estragole and 1-hexanol. These results suggested that volatile compounds were co-regulated according to specific modules within the F_1_ population.

### QTL mapping for volatile compounds in ‘Fuji’ × ‘Cripps Pink’

With the combination of the phenotypic data and the constructed genetic linkage map, the QTLs underlying volatile compounds in the F_1_ progeny fruits of ‘Fuji’ × ‘Cripps Pink’ population were identified for two years. As shown in **Table 3**, a total of 87 QTLs were detected for 15 volatiles on 14 linkage groups (LG1, LG2, LG3, LG4, LG6, LG7, LG8, LG9, LG10, LG11, LG12, LG15, LG16, LG17). The phenotypic variance (R^2^) explained by these QTLs ranged from 5.1% (qHex19-1) to 23.1% (qHex19-4) with the LOD value varied from 3.05 to 10.34. Among them, 25 QTLs for 4 esters (hexyl acetate, hexyl butanoate, hexyl 2-methylbutyrate and hexyl hexanoate) mapped on LG2, LG4, LG6, LG8, LG11, LG15, LG17 were identified and explained the phenotypic variation from 6.0% to 23.1% (**Figure S2**). As for 2 alcohols (2-methyl-1-butanol, 1-hexanol) and 5 aldehydes (hexanal, (*E*)-2-hexenal, nonanal, (*E*)-2-octenal and (*Z*)-2-nonenal), 12 and 29 QTLs were detected on 5 and 13 linkage groups, respectively (Figures S3 and S4). A total of 21 QTLs controlling 4 other volatiles (pentylcyclopropane, estragole, α-farnesene and (*Z*)-anethole) were identified on 9 linkage groups with the LOD value from 3.15 to 12.06 (**Figure S5**). Moreover, QTLs for hexyl butanoate (qBut19-1, qBut20-2), QTLs for hexyl hexanoate (qHex19-2, qHex20-2) and another QTLs for hexyl 2-methylbutyrate (qMet19-1, qMet20-1), identified on LG6 with the LOD peak value 8.70, 8.55, 4.00, 6.71, 5.48 and 7.65, explaining the maximum phenotypic variation 16.7%, 16.5%, 9.7% 13.5%, 9.9% and 14.8%, fell in the same chromosomal region in two consecutive years. Notably, a QTL controlling hexyl acetate, namely qAce19-2, with the LOD peak value 3.18, explaining the maximum phenotypic variation 6.6% was positioned within the same physical interval on LG6. These QTLs about four ester compounds found on LG6 were overlapping, indicating that this was critical for the biosynthesis of esters.

**Table 3:** Summary of QTLs identified for 15 volatile compounds in F_1_ progeny fruits of ‘Fuji’ × ‘Cripps Pink’ population for two years.

Volatile compound	QTL	Chr	Year	Interval (cM)	Start marker	End marker	LOD_max_	R^2^ (%)
Hexyl acetate	qAce19-1	2	2019	5.04–7.86	Block 530	Block 570	4.77	9.8
	qAce19-2	6	2019	7.92–9.27	Block 1445	Block 1452	3.18	6.6
	qAce19-3	15	2019	71.32–72.83	Block 4192	Block 4200	3.83	8.0
	qAce20-1	2	2020	5.13–7.86	Block 542	Block 570	6.55	13.2
	qAce20-2	8	2020	47.25–49.46	Block 2056	Block 2077	4.13	8.5
	qAce20-3	15	2020	80.12–92.52	Block 4205	Block 4274	4.54	9.3
Hexyl butanoate	qBut19-1	6	2019	7.42–9.27	Block 1443	Block 1452	8.70	16.7
	qBut19-2	15	2019	84.44–87.64	Block 4250	Block 4265	5.44	10.2
	qBut20-1	4	2020	85.17–86.44	Block 1008	Block 1020	4.12	8.3
	qBut20-2	6	2020	8.09–9.27	Block 1446	Block 1452	8.55	16.5
	qBut20-3	15	2020	84.45–86.12	Block 4251	Block 4260	6.42	13.4
Hexyl 2-methylbutyrate	qMet19-1	6	2019	7.42–8.93	Block 1443	Block 1450	5.48	9.9
	qMet19-2	8	2019	54.74–55.93	Block 2021	Block 2026	3.26	6.0
	qMet19-3	11	2019	42.52–45.06	Block 3032	Block 3037	3.13	5.8
	qMet19-4	15	2019	78.56–81.25	Block 4222	Block 4233	4.69	8.5
	qMet20-1	6	2020	7.42–9.27	Block 1443	Block 1452	7.65	14.8
	qMet20-2	15	2020	79.74–83.77	Block 4226	Block 4247	4.72	9.4
Hexyl hexanoate	qHex19-1	4	2019	38.12–39.75	Block 970	Block 976	3.05	5.1
	qHex19-2	6	2019	7.42–9.27	Block 1443	Block 1452	4.00	9.7
	qHex19-3	11	2019	77.71–82.92	Block 2915	Block 2944	4.02	9.9
	qHex19-4	17	2019	130.29–131.47	Block 4762	Block 4769	10.34	23.1
	qHex20-1	2	2020	53.73–61.88	Block 453	Block 459	3.63	7.6
	qHex20-2	6	2020	7.42–9.27	Block 1443	Block 1452	6.71	13.5
	qHex20-3	15	2020	81.25–83.77	Block 4233	Block 4247	4.82	9.9
	qHex20-4	17	2020	11.29–13.98	Block 4964	Block 4978	3.45	7.2
2-Methyl-1-butanol	qButa19-1	2	2019	108.40–113.39	Block 297	Block 302	7.81	14.1
	qButa19-2	4	2019	83.25–85.37	Block 1005	Block 1015	6.60	8.2
	qButa19-3	15	2019	123.75–125.94	Block 4386	Block 4396	5.35	9.9
	qButa20-1	2	2020	110.54–113.39	Block 299	Block 302	11.92	22.2
	qButa20-2	15	2020	123.75–133.10	Block 4390	Block 4421	11.30	14.6
1-Hexanol	qHexa19-1	2	2019	108.40–110.12	Block 297	Block 298	5.04	9.1
	qHexa19-2	3	2019	81.05–84.26	Block 728	Block 735	5.29	9.6
	qHexa19-3	15	2019	70.82–73.68	Block 4189	Block 4204	4.51	7.2
	qHexa19-4	17	2019	20.54–23.12	Block 4957	Block 4966	4.62	8.9
	qHexa20-1	2	2020	95.65–97.17	Block 347	Block 353	4.24	8.5
	qHexa20-2	2	2020	108.40–113.39	Block 297	Block 302	4.70	9.4
	qHexa20-3	17	2020	57.69–58.03	Block 4917	Block 4921	4.63	9.2
Hexanal	qAna19-1	1	2019	114.85–116.71	Block 232	Block 239	8.72	15.3
	qAna19-2	7	2019	13.97–14.81	Block 1766	Block 1771	4.11	7.5
	qAna19-3	12	2019	65.04–66.22	Block 3348	Block 3353	5.77	10.4
	qAna20-1	1	2020	114.86–116.55	Block 232	Block 238	6.22	12.2
	qAna20-2	7	2020	2.86–3.71	Block 1713	Block 1715	3.55	7.2
	qAna20-3	9	2020	24.00–25.01	Block 2387	Block 2393	3.14	6.4
	qAna20-3	16	2020	108.39–108.90	Block 4588	Block 4590	3.32	6.7
(*E*)-2-Hexenal	qEna19-1	1	2019	110.64–111.98	Block 219	Block 224	3.96	7.3
	qEna19-2	7	2019	1.01–2.36	Block 1706	Block 1710	3.29	6.1
	qEna19-3	9	2019	89.04–90.39	Block 2256	Block 2262	3.77	6.9
	qEna19-4	15	2019	96.74–100.98	Block 4299	Block 4306	3.72	6.8
	qEna20-1	1	2020	110.47–115.70	Block 218	Block 233	6.13	12.0
Nonanal	qNon19-1	3	2019	119.31–127.34	Block 854	Block 871	3.84	7.6
	qNon20-1	3	2020	23.16–24.17	Block 638	Block 644	3.98	8.2
	qNon20-2	4	2020	39.47–39.64	Block 929	Block 930	3.93	8.1
(*E*)-2-Octenal	qOct19-1	2	2019	81.18–83.03	Block 410	Block 417	4.53	10.3
	qOct19-2	12	2019	13.18–15.71	Block 3191	Block 3202	5.43	12.2
	qOct19-3	15	2019	71.66–74.69	Block 4194	Block 4206	3.38	7.8
	qOct19-4	16	2019	185.74–187.73	Block 4455	Block 4456	3.13	7.2
	qOct20-1	1	2020	112.32–115.87	Block 225	Block 234	4.09	8.2
	qOct20-2	4	2020	50.34–51.01	Block 983	Block 987	7.89	15.3
(*Z*)-2-Nonenal	qNone19-1	2	2019	96.50–98.18	Block 343	Block 351	3.59	11.6
	qNone19-2	6	2019	145.64–146.48	Block 1569	Block 1574	3.03	9.9
	qNone19-3	12	2019	37.64–38.65	Block 3239	Block 3244	3.72	12.0
	qNone19-4	15	2019	65.44–67.12	Block 4162	Block 4171	4.42	14.1
	qNone20-1	4	2020	50.34–51.01	Block 983	Block 987	3.17	6.5
	qNone20-2	10	2020	142.75–143.93	Block 2512	Block 2517	3.27	6.6
	qNone20-3	11	2020	42.52–43.02	Block 3034	Block 3037	3.16	6.4
	qNone20-4	11	2020	68.94–71.46	Block 2945	Block 2957	3.24	6.6
Pentylcyclopropane	qPen19-1	7	2019	29.91–31.26	Block 1802	Block 1806	3.26	9.7
	qPen19-2	15	2019	112.27–113.28	Block 4347	Block 4352	3.15	9.4
	qPen19-3	16	2019	85.93–88.81	Block 4648	Block 4652	3.74	11.1
	qPen20-1	15	2020	111.76–118.83	Block 4345	Block 4380	3.58	7.4
	qPen20-2	15	2020	141.08–142.43	Block 4437	Block 4445	3.51	7.3
Estragole	qEst19-1	3	2019	43.74–47.64	Block 710	Block 718	5.99	10.9
	qEst19-2	3	2019	98.78–103.17	Block 755	Block 771	6.04	11.0
	qEst19-3	16	2019	140.32–143.20	Block 4521	Block 4529	5.75	10.5
	qEst19-4	17	2019	130.29–131.47	Block 4759	Block 4769	4.45	8.2
	qEst20-1	3	2020	98.61–103.17	Block 754	Block 771	12.06	22.4
α-Farnesene	qFar19-1	4	2019	58.60–59.61	Block 988	Block 992	7.73	14.7
	qFar19-2	12	2019	8.58–8.75	Block 3183	Block 3184	8.05	15.2
	qFar19-3	15	2019	95.56–100.14	Block 4295	Block 4305	6.72	12.9
	qFar20-1	8	2020	37.22–37.89	Block 2092	Block 2094	3.51	7.1
	qFar20-2	15	2020	96.74–102.67	Block 4299	Block 4311	8.10	15.6
(*Z*)-Anethole	qAne19-1	3	2019	44.59–44.76	Block 711	Block 712	3.86	7.5
	qAne19-2	15	2019	84.45–88.82	Block 4250	Block 4270	4.18	8.1
	qAne19-3	17	2019	130.29–131.47	Block 4759	Block 4769	4.60	8.9
	qAne20-1	3	2020	33.93–35.95	Block 687	Block 697	4.64	9.4
	qAne20-2	3	2020	98.61–109.71	Block 754	Block 771	4.83	9.8
	qAne20-3	11	2020	79.89–81.24	Block 2924	Block 2932	4.21	8.6

R^2^, Percentage of the total phenotypic variation explained by the QTL.

### Identification and function verification of candidate genes

**Figure 3:**
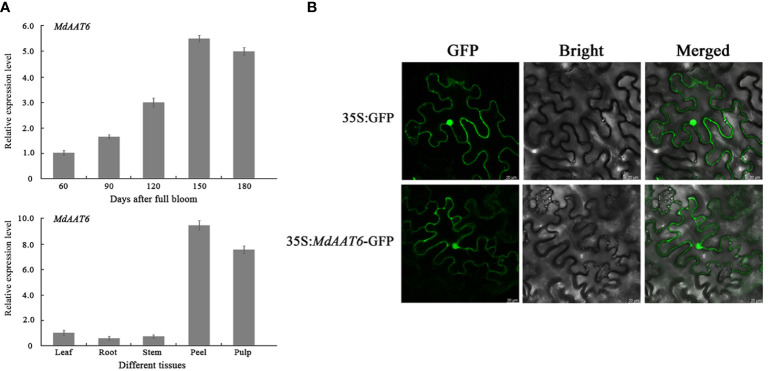
Expression pattern analysis of the candidate gene. **(A)** Expression levels of *MdAAT6* at different development stages and tissues in ‘Fuji’ apple fruit. **(B)** Subcellular localization of MdAAT6 in tobacco leaves.

**Figure 4:**
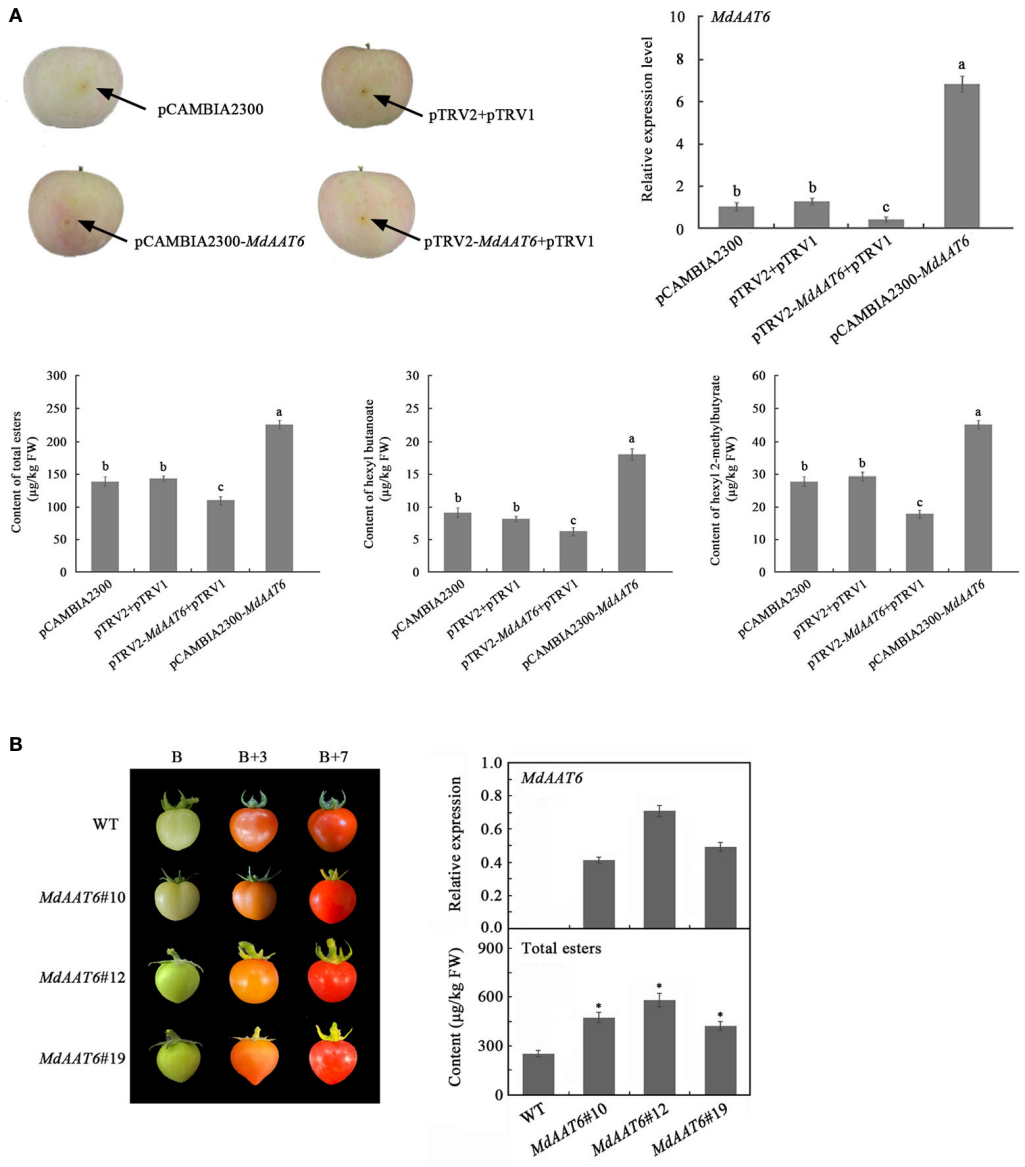
Function verification of the candidate gene. **(A)** Transient over-expression and silent expression of *MdAAT6* in apple fruit. **(B)** Over-expression of *MdAAT6* in ‘Micro-Tom’ tomato. The symbol "*" and small letters indicate significant differences between samples.

To identify putative candidate genes involved in apple fruit ester synthesis, the QTLs corresponding to ester compounds on LG6 were further analyzed. Based on the annotation of the apple reference genome (Daccord et al., 2017) and transcriptome sequencing results of the parents (Liu et al., 2021), the differentially expression genes that fell within the QTL mapping interval were scanned, and one candidate gene (MD06G1016500) was screened. The AAT gene (MD06G1016500, named *MdAAT6*) located on LG6 showed the high transcript levels in this QTL. Therefore, we further investigated the role of *MdAAT6* in influencing volatile ester formation. As shown in **Figure 3A**, with the development of apple fruit, the transcript level of *MdAAT6* continued to increase and showed highest expression levels at 150 days after full bloom, about 5.5-fold compared to 60 days after full bloom. The tissue-specificity of *MdAAT6* expression indicated that its transcript significantly accumulated in peel and pulp (more than 8.0-fold compared to leaf, root and stem) at the ripening stage. In order to determine the subcellular location of MdAAT6, the recombinant GFP fusion protein was transiently overexpressed in tobacco leaves and the green fluorescence signal indicated that MdAAT6 was located in the cytoplasm (**Figure 3B**). Furthermore, as shown in **Figure 4A**, transient over-expression of *MdAAT6* in apple fruit significantly increased the hexyl butanoate (7.38 μg/kg FW), hexyl 2-methylbutyrate (43.47 μg/kg FW) production and hence, esters accumulation (225.82 μg/kg FW), while silencing the expression of *MdAAT6* inhibited the production of ester compounds. To verify the regulatory effects of candidate gene in ester synthesis, over-expression of *MdAAT6* in ‘Micro-Tom’ tomato was conducted. Three transgenic lines were generated, with no differences in fruit size or color between the wild type and transgenic tomato plants. Compared with the wild type, the esters contents in transgenic tomato fruit were significantly higher (approximately 1.80-fold) (**Figure 4B**). Taken together, these results suggest that the candidate gene *MdAAT6* contributes to esters accumulation in apple fruit.

## Discussion

Aroma, which is generally a complex mixture of volatile compounds, has a great influence on the sensory attributes and overall flavour for apple fruit (Aprea et al., 2012; Yang et al., 2021a). The volatile profiles of flavour compounds in apple fruit have been investigated extensively in previous studies involving single cultivars, as well as in pairwise comparisons or based on a few different varieties (Altisent et al., 2009; Yan et al., 2020; Yang et al., 2022). In recent years, aroma profiling has also been carried out in apple progenies targeting the genomic regions that might regulate the volatile compound production. Dunemann et al. (2009a) measured the volatile compounds of a cross between the apple cultivars ‘Discovery’ and ‘Prima’, consisting of 150 F_1_ plants. The results showed that some compounds such as butanol, butyl butanoate, pentyl acetate and hexyl acetate exhibited a continuous variation in the progeny, which was typical for a polygenic inheritance, and transgressive segregation occurred in both directions. Rowan et al. (2009) reported the concentrations of most volatiles in ‘Royal Gala’ × ‘Granny Smith’ apple cross a continuous distribution of values, but the segregation ratio for 2-methylbutyl acetate differed slightly from a 1:1 ratio, suggestive of control by a single major gene. Costa et al. (2013) investigated volatile organic compound (VOC) variability in the ‘Fiesta’ × ‘Discovery’ population over three different environments and the observation showed phenotypic variability assessed in the seedlings largely exceeds the two parental cultivars, suggesting a transgressive segregation in this progeny. In our work, the parents ‘Fuji’ and ‘Cripps Pink’ not only showed different volatile patterns, but also it was evident, that in ‘Cripps Pink’ the absolute volatile concentrations were higher for the majority of esters. In the progeny, a total of 15 compounds were detected such as α-farnesene, 1-butanol and hexyl acetate and showed continuous variation, typical of polygenic inheritance, although their distributions were generally skewed toward high values, in accordance with previous research (Dunemann et al., 2009a). The single volatile compounds exhibited different frequency distributions in the progeny, indicating diverse modes of aroma inheritance. Overall, these results suggested the genetic effect largely contributes to the general apple aroma phenotypic variance.

QTL mapping has been widely used in genetic studies to identify genomic regions potentially associated to the target traits. In the previous study, Dunemann et al. (2009a) used a saturated molecular linkage map of ‘Discovery’ × ‘Prima’ apple to identify QTLs for aroma compounds and a total of 50 QTLs for 27 volatiles were obtained on 12 apple chromosomes through interval mapping, which mainly clustered on linkage groups LG 2, 3 and 9. Costa et al. (2013) combined the QTL and volatile organic compounds analysis on the progeny ‘Fiesta’ × ‘Discovery’ to validate the presence of important QTLs in three specific genomic regions, located on the linkage group 2 and 15. Souleyre et al. (2014) investigated the phenotypic variability in ester accumulation with the population from the ‘Royal Gala’ and ‘Granny Smith’ cross and 46 QTLs for the production of ester and alcohol compounds were identified on 15 linkage groups, with the major QTL of 35 compounds positioned on LG2. Moreover, Cappellin et al., 2015b conducted the comprehensive QTL survey about volatile organic compounds in the population ‘Fuji’ × ‘Delearly’ and the coincident location between a group of QTLs on chromosome 2 was verified, mainly related to esters and alcohols. In our study, we effectively employed GC-MS for studying the genetic control of aroma emission in apple progeny and a classical QTL investigation related to the volatile production was applied. The mapping result of one QTL related to hexyl acetate (qAce19-1) was located on the LG 2, in agreement with that the major QTL for ester compounds was positioned on LG 2 in other studies (Souleyre et al., 2014; Cappellin et al., 2015b). However, it is worth noting that a QTL mapped on linkage group 6 was found to be probably associated with ester production. In that QTL mapping interval, combined with the transcriptome sequencing data, an *AAT* candidate gene was positioned and confirmed. Additionally, for alcohol and aldehyde compounds QTL detection, many QTL loci have been mined in our study and further work need to be done to better dissect the genetic determinants impacting this trait.

The ester compounds in apple fruit are thought to be produced from two main different pathways, the fatty acid pathway for formation of straight chain esters and the isoleucine pathway contributing to branched-chain esters (Rowan et al., 1999). Alcohol acyl-transferase (AAT) belonging to the BAHD superfamily is the key enzyme involved in the last step of ester biosynthesis, which catalyze the transfer of an acyl group from a coenzyme A (CoA) donor to an alcohol acceptor (D’Auria, 2006; Souleyre et al., 2014). In the past few years, some *AAT* genes have been isolated and studied in lots of fruits, including banana (Beekwilder et al., 2004), melon (Lucchetta et al., 2007), kiwifruit (Gunther et al., 2011), strawberry (Cumplido-Laso et al., 2012) and peach (Song et al., 2021). Meanwhile, there have been a few *AAT* genes identified and characterized in apple fruit for their putative biochemical functions and expression patterns (Zhu et al., 2008). The *MdAAT1* gene was proved to participate in the production of the main ester compounds (hexyl acetate, butyl acetate and 2-methyl-butyl acetate) in ‘Royal Gala’ apple fruit (Souleyre et al., 2005). Moreover, four single nucleotide polymorphisms (SNPs) of *MdAAT1* related to variation in ester production were identified, the 468-bp region was used to screen a set of apple cultivars for association the level of esters (Dunemann et al., 2012). The highly homologous gene *MdAAT2* isolated from ‘Golden Delicious’ apple was also found to be positively correlated with AAT enzyme activity and ester formation (Li et al., 2006). Furthermore, Zhu et al. (2008) reported that in ‘Golden Delicious’ and ‘Granny Smith’ apples, the expression levels of both *MdAAT1* and *MdAAT2* genes increased with fruit ripening stages, which consistent with the total ester content accumulation pattern. In our study, the association of a candidate gene (*MdAAT6*) with esters production was validated by QTL analysis and candidate gene mapping in a segregating population obtained from the ‘Fuji’ × ‘Cripps Pink’. Transient transformation of apple fruits and transgenic tomato plants with enhanced *MdAAT6* expression produced more esters. Functional characterization demonstrated that the candidate gene *MdAAT6* could catalyze the synthesis of esters and produce high levels in ripe fruit, suggesting *MdAAT6* also one of the candidate genes responsible for apple fruit ester production besides *MdAAT1* and *MdAAT2*.

## Conclusion

In this work, volatile profiles were evaluated by GC-MS over 2019 and 2020 in ripe fruit of F_1_ population between ‘Fuji’ and ‘Cripps Pink’ and the genetics underlying aroma compounds was dissected. Moreover, a total of 87 QTLs were detected for 15 volatile compounds on 14 linkage groups. Among them, a set of QTL associated with ester production was identified and confirmed on LG 6. The candidate gene *MdAAT6* in the QTL mapping interval was shown to be critical for the biosynthesis of esters. Knowledge on the regulation of volatile synthesis pathways will greatly facilitate studies to improve apple fruit flavor quality. Our findings provided the ground for the molecular mechanism of volatile compounds formation and new insights for apple flavor improvement in the future.

## Data availability statement

The datasets presented in this study can be found in online repositories. The names of the repository/repositories and accession number(s) can be found in the text.

## Author contributions

YS and ZZ designed the study. YJ analyzed the data. YH supervised the project. YS drafted the manuscript. ZZ gave critical suggestions throughout the study and revised the manuscript. All authors contributed to the article and approved the submitted version.

## References

[B1] Altisent R. Echeverria G. Graell J. Lopez L. Lara I. (2009). Lipoxygenase activity is involved in the regeneration of volatile ester-synthesizing capacity after ultra-low oxygen storage of ‘Fuji’ apple. J. Agric. Food Chem. 57, 4305–4312. doi: 10.1021/jf803930j 19378945

[B2] Aprea E. Corollaro M. Betta E. Endrizzi I. Dematte M. Biasioli F.. (2012). Sensory and instrumental profiling of 18 apple cultivars to investigate the relation between perceived quality and odour and flavour. Food Res. Int. 49, 677–686. doi: 10.1016/j.foodres.2012.09.023

[B3] Ban Y. Oyama-Okubo N. Honda C. Nakayama M. Moriguchi T. (2010). Emitted and endogenous volatiles in ‘Tsugaru’ apple: The mechanism of ester and (*E*, *E*)-α-farnesene accumulation. Food Chem. 118, 272–277. doi: 10.1016/j.foodchem.2009.04.109

[B4] Beekwilder J. Alvarez-Huerta M. Neef E. Verstappen F. Bouwmeester H. Aharoni A. (2004). Functional characterization of enzymes forming volatile esters from strawberry and banana. Plant Physiol. 135, 1865–1878. doi: 10.1104/pp.104.042580 15326278 PMC520758

[B5] Cappellin L. Costa F. Aprea E. Betta E. Gasperi F. Biasioli F. (2015a). Double clustering of PTR-ToF-MS data enables the mapping of QTLs related to apple fruit volatilome. Sci. Hortic. 197, 24–32. doi: 10.1016/j.scienta.2015.10.043

[B6] Cappellin L. Farneti B. Di G. Busatto N. Khomenko I. Romano A.. (2015b). QTL analysis coupled with PTR-ToF-MS and candidate gene-based association mapping validate the role of *MdAAT1* as a major gene in the control of flavor in apple fruit. Plant Mol. Biol. Rep. 33, 239–252. doi: 10.1007/s11105-014-0744-y

[B7] Chen P. Li Z. Zhang D. Shen W. Xie Y. Zhang J.. (2021). Insights into the effect of human civilization on *Malus* evolution and domestication. Plant Biotechnol. J. 19, 2206–2220. doi: 10.1111/pbi.13648 34161653 PMC8541786

[B8] Costa F. Cappellin L. Zini E. Patocchi A. Kellerhals M. Komjanc M.. (2013). QTL validation and stability for volatile organic compounds (VOCs) in apple. Plant Sci. 211, 1–7. doi: 10.1016/j.plantsci.2013.05.018 23987805

[B9] Cumplido-Laso G. Medina-Puche L. Moyano E. Hoffmann T. Sinz Q. Ring L.. (2012). The fruit ripening-related gene *FaAAT2* encodes an acyl transferase involved in strawberry aroma biogenesis. J. Exp. Bot. 63, 4275–4290. doi: 10.1093/jxb/ers120 22563120

[B10] Daccord N. Celton J. Linsmith G. Becker C. Choisne N. Schijlen E.. (2017). High-quality *de novo* assembly of the apple genome and methylome dynamics of early fruit development. Nat. Genet. 49, 1099–1106. doi: 10.1038/ng.3886 28581499

[B11] D’Auria J. (2006). Acyltransferases in plants: A good time to be BAHD. Curr. Opin. Plant Biol. 9, 331–340. doi: 10.1016/j.pbi.2006.03.016 16616872

[B12] Dixon J. Hewett E. (2000). Factors affecting apple aroma/flavour volatile concentration: A review. N. Z J. Crop Hortic. Sci. 28, 155–173. doi: 10.1080/01140671.2000.9514136

[B13] Dunemann F. Boudichevskaia A. Grafe C. Weber W. Ulrich D. (2009b). QTL and candidate gene mapping for aroma compounds in the apple progeny discovery × prima. Acta Hortic. 839, 433–440. doi: 10.17660/ActaHortic.2009.839.58

[B14] Dunemann F. Ulrich D. Boudichevskaia A. Grafe C. Weber E. (2009a). QTL mapping of aroma compounds analysed by headspace solid-phase microextraction gas chromatography in the apple progeny ‘Discovery’ × ‘Prima’. Mol. Breeding 23, 501–521. doi: 10.1007/s11032-008-9252-9

[B15] Dunemann F. Ulrich D. Malysheva-Otto L. Weber W. Longhi S. Velasco R.. (2012). Functional allelic diversity of the apple alcohol acyl-transferase gene *MdAAT1* associated with fruit ester volatile contents in apple cultivars. Mol. Breeding 29, 609–625. doi: 10.1007/s11032-011-9577-7

[B16] Elss S. Preston C. Appel M. Heckel F. Schreier P. (2006). Influence of technological processing on apple aroma analysed by high resolution gas chromatographymass spectrometry and on-line gas chromatography-combustion/pyrolysis-isotope ratio mass spectrometry. Food Chem. 98, 269–276. doi: 10.1016/j.foodchem.2005.06.011

[B17] Espino-Diaz M. Sepulveda D. Gonzalez-Aguilar G. Olivas G. (2016). Biochemistry of apple aroma: A review. Food Technol. Biotechnol. 54, 375–394. doi: 10.17113/ftb.54.04.16.4248 28115895 PMC5253989

[B18] Gunther C. Chervin C. Marsh K. Newcomb R. Souleyre E. (2011). Characterisation of two alcohol acyltransferases from kiwifruit (*Actinidia* spp.) reveals distinct substrate preferences. Phytochemistry 72, 700–710. doi: 10.1016/j.phytochem.2011.02.026 21450321

[B19] Hao Y. Hu G. Breitel D. Liu M. Mila I. Frasse P.. (2015). Auxin response factor SlARF2 is an essential component of the regulatory mechanism controlling fruit ripening in tomato. PloS Genet. 11, e1005649. doi: 10.1371/journal.pgen.1005649 26716451 PMC4696797

[B20] Klee H. (2010). Improving the flavor of fresh fruits: Genomics, biochemistry, and biotechnology. New Phytol. 187, 44–56. doi: 10.1111/j.1469-8137.2010.03281.x 20456053

[B21] Li D. Xu Y. Xu G. Gu L. Li D. Shu H. (2006). Molecular cloning and expression of a gene encoding alcohol acyltransferase (*MdAAT2*) from apple (cv. golden delicious). Phytochemistry 67, 658–667. doi: 10.1016/j.phytochem.2006.01.027 16524607

[B22] Liu L. (2022). Identification of QTLs for fruit texture and color traits in apple (Malus × domestica borkh.). [dissertation’s thesis] (Yangling: Northwest A&F University).

[B23] Liu X. Hao N. Feng R. Meng Z. Li Y. Zhao Z. (2021). Transcriptome and metabolite profiling analyses provide insight into volatile compounds of the apple cultivar ‘Ruixue’ and its parents during fruit development. BMC Plant Biol. 21, 231. doi: 10.1186/s12870-021-03032-3 34030661 PMC8147058

[B24] Lucchetta L. Manriquez D. El-Sharkawy I. Flores F. Sanchez-Bel P. Zouine M.. (2007). Biochemical and catalytic properties of three recombinant alcohol acyltransferases of melon. sulfur-containing ester formation, regulatory role of CoA-SH in activity, and sequence elements conferring substrate preference. J. Agric. Food Chem. 55, 5213–5220. doi: 10.1021/jf070210w 17542607

[B25] Mehinagic E. Royer G. Symoneaux R. Jourjon F. Prost C. (2006). Characterization of odor-active volatiles in apples: Influence of cultivars and maturity stage. J. Agric. Food Chem. 54, 2678–2687. doi: 10.1021/jf052288n 16569061

[B26] Rowan D. Allen J. Fielder S. Hunt B. (1999). Biosynthesis of straight-chain ester volatiles in red delicious and granny smith apples using deuterium-labelled precursors. J. Agric. Food Chem. 47, 2553–2562. doi: 10.1021/jf9809028 10552526

[B27] Rowan D. Hunt M. Dimouro A. Alspach P. Chagne D. (2009). Profiling fruit volatiles in the progeny of a ‘Royal gala’ × ‘Granny smith’ apple (*Malus* × *domestica*) cross. J. Agric. Food Chem. 57, 7953–7961. doi: 10.1021/jf901678v 19691320

[B28] Sainsbury F. Thuenemann E. Lomonossoff G. (2009). pEAQ: versatile expression vectors for easy and quick transient expression of heterologous proteins in plants. Plant Biotechnol. J. 7, 682–693. doi: 10.1111/j.1467-7652.2009.00434.x 19627561

[B29] Schaffer R. Friel E. Souleyre E. Bolitho K. Thodey K. Ledger S.. (2007). A genomics approach reveals that aroma production in apple is controlled by ethylene predominantly at the final step in each biosynthetic pathway. Plant Physiol. 144, 1899–1912. doi: 10.1104/pp.106.093765 17556515 PMC1949883

[B30] Song J. Forney C. (2008). Flavour volatile production and regulation in fruit. Can. J. Plant Sci. 88, 537–550. doi: 10.4141/CJPS07170

[B31] Song Z. Peng B. Gu Z. Tang M. Li B. Liang M.. (2021). Site-directed mutagenesis identified the key active site residues of alcohol acyltransferase *PpAAT1* responsible for aroma biosynthesis in peach fruits. Hortic. Res. 8, 32. doi: 10.1038/s41438-021-00461-x 33518702 PMC7847995

[B32] Souleyre E. Chagne D. Chen X. Tomes S. Turner R. Wang M.. (2014). The *AAT1* locus is critical for the biosynthesis of esters contributing to ‘ripe apple’ flavour in ‘Royal gala’ and ‘Granny smith’ apples. Plant J. 78, 903–915. doi: 10.1111/tpj.12518 24661745

[B33] Souleyre E. Greenwood D. Friel E. Karunairetnam S. Newcomb R. (2005). An alcohol acyl transferase from apple (cv. royal gala), *MpAAT1*, produces esters involved in apple fruit flavor. FEBS J. 272, 3132–3144. doi: 10.1111/j.1742-4658.2005.04732.x 15955071

[B34] Xu Y. Lu Y. Xie C. Gao S. Wan J. Prasanna B. (2012). Whole-genome strategies for marker-assisted plant breeding. Mol. Breeding 29, 833–854. doi: 10.1007/s11032-012-9699-6

[B35] Yan D. Shi J. Ren X. Tao Y. Ma F. Li R.. (2020). Insights into the aroma profiles and characteristic aroma of ‘Honeycrisp’ apple (*Malus* × *domestica*). Food Chem. 327, 127074. doi: 10.1016/j.foodchem.2020.127074 32464463

[B36] Yang S. Hao N. Meng Z. Li Y. Zhao Z. (2021b). Identification, comparison and classification of volatile compounds in peels of 40 apple cultivars by HS-SPME with GC-MS. Foods 10, 1051. doi: 10.3390/foods10051051 34064741 PMC8151858

[B37] Yang S. Li D. Li S. Yang H. Zhao Z. (2022). GC-MS metabolite and transcriptome analyses reveal the differences of volatile synthesis and gene expression profiling between two apple varieties. Int. J. Mol. Sci. 23, 2939. doi: 10.3390/ijms23062939 35328360 PMC8951106

[B38] Yang S. Meng Z. Fan J. Yan L. Yang Y. Zhao Z. (2021a). Evaluation of the volatile profiles in pulp of 85 apple cultivars (*Malus domestica*) by HS-SPME combined with GC-MS. J. Food Meas. Charact. 15, 4215–4225. doi: 10.1007/s11694-021-01003-8

[B39] Zhu D. Ren X. Wei L. Cao X. Ge Y. Liu H.. (2020). Collaborative analysis on difference of apple fruits flavour using electronic nose and electronic tongue. Sci. Hortic. 260, 108879. doi: 10.1016/j.scienta.2019.108879

[B40] Zhu Y. Rudell D. Mattheis J. (2008). Characterization of cultivar differences in alcohol acyltransferase and 1-aminocyclopropane-1-carboxylate synthase gene expression and volatile ester emission during apple fruit maturation and ripening. Postharvest Biol. Technol. 49, 330–339. doi: 10.1016/j.postharvbio.2008.03.015

[B41] Zini E. Biasioli F. Gasperi F. Mott D. Aprea E. Mark T.. (2005). QTL mapping of volatile compounds in ripe apples detected by proton transfer reaction-mass spectrometry. Euphytica 145, 269–279. doi: 10.1007/s10681-005-1645-9

