# Supplementary material for: Genetic analysis and QTL mapping of aroma volatile compounds in the apple progeny ‘Fuji’ × ‘Cripps Pink’
Source: Front Plant Sci. 2023 Mar 20;14:1048846. doi: 10.3389/fpls.2023.1048846 (PMC10067597; doi:10.3389/fpls.2023.1048846)
Supplement: Supplementary file 1 [file DataSheet_1.doc]

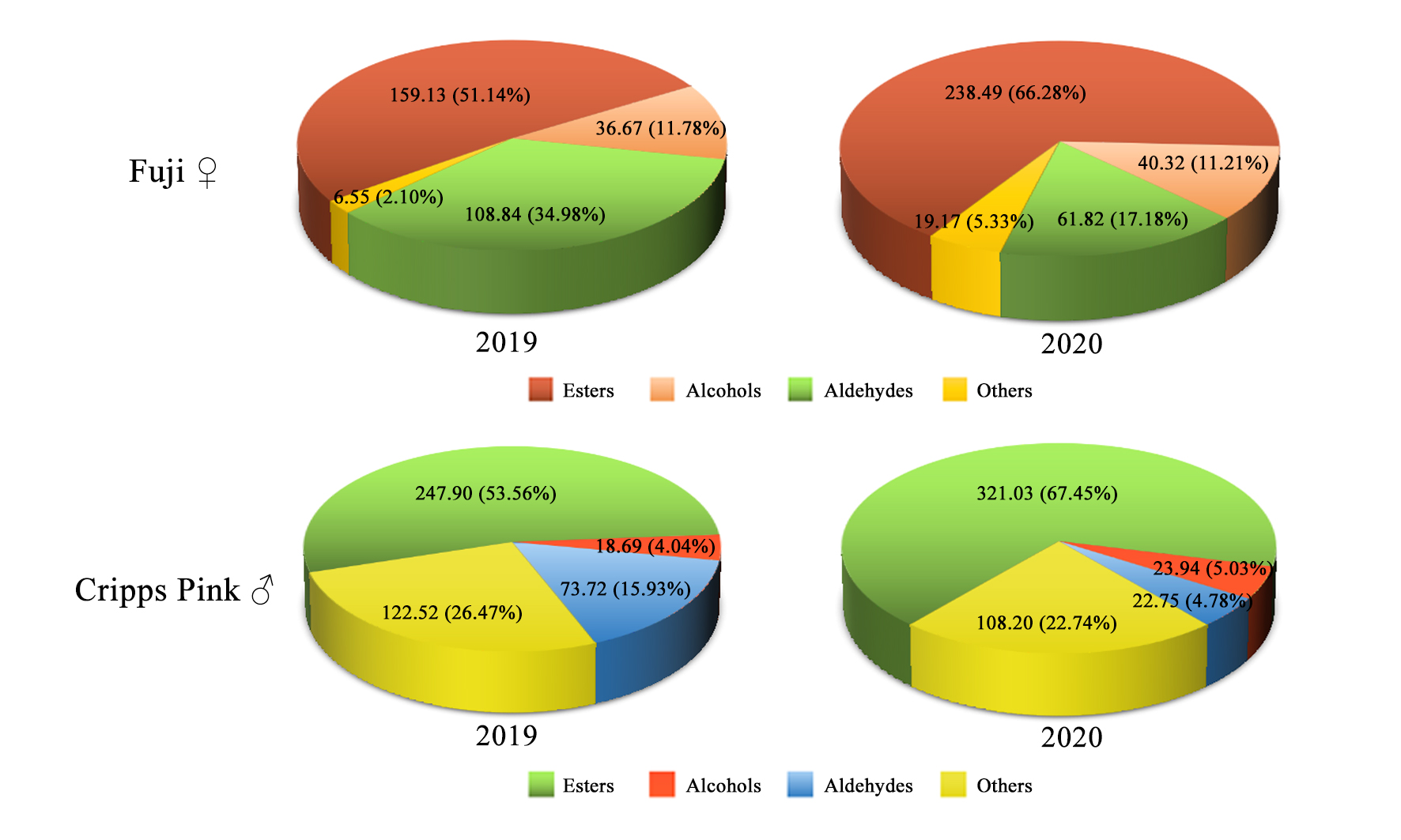


Figure S1 The total content (μg/kg FW) and percentage (%) of different types of volatile compounds in parent fruits.


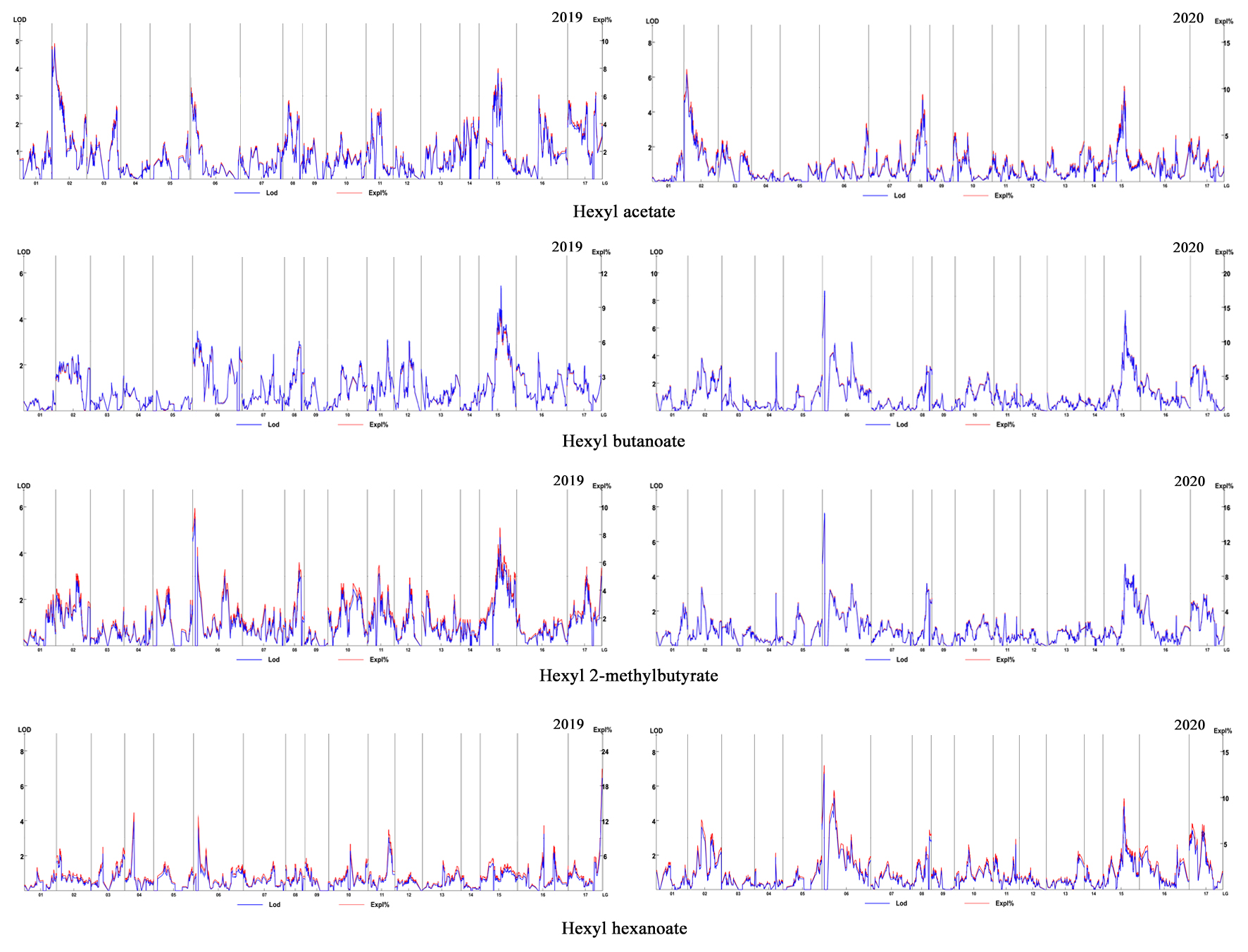


Figure S2 QTL mapping of four ester compounds in 2019 and 2020


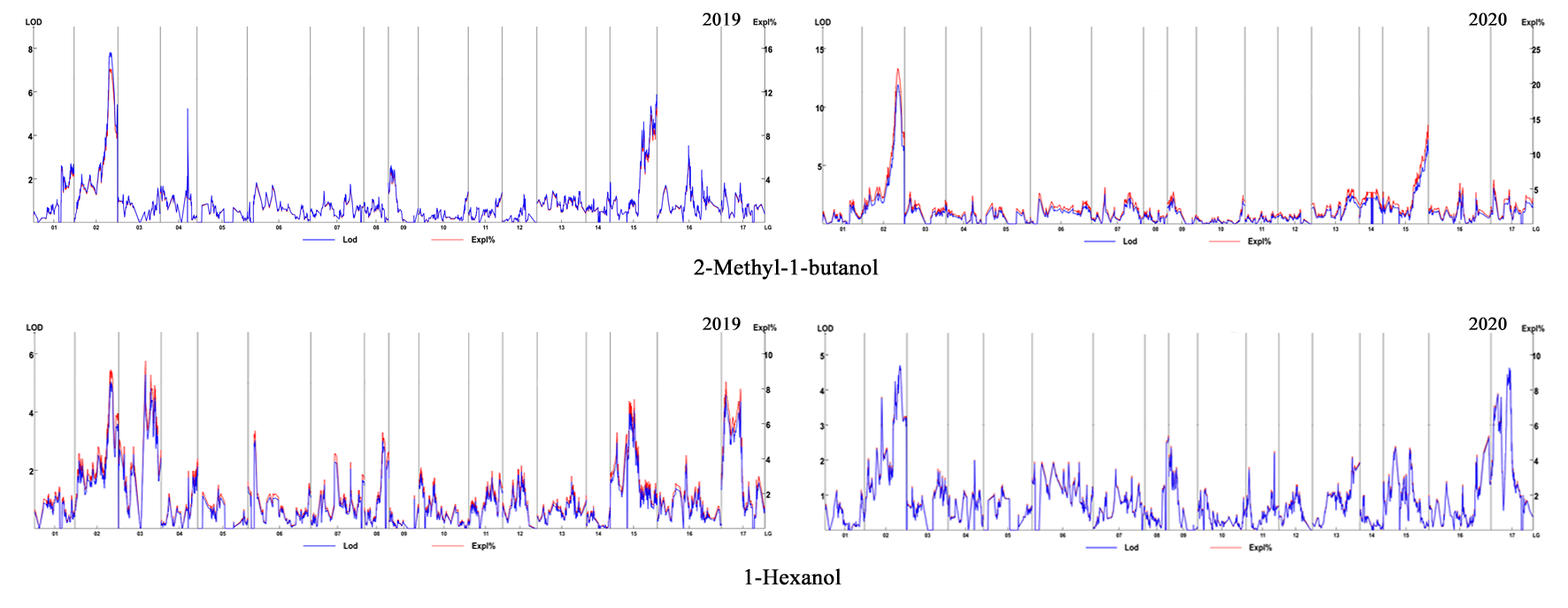


Figure S3 QTL mapping of two alcohol compounds in 2019 and 2020


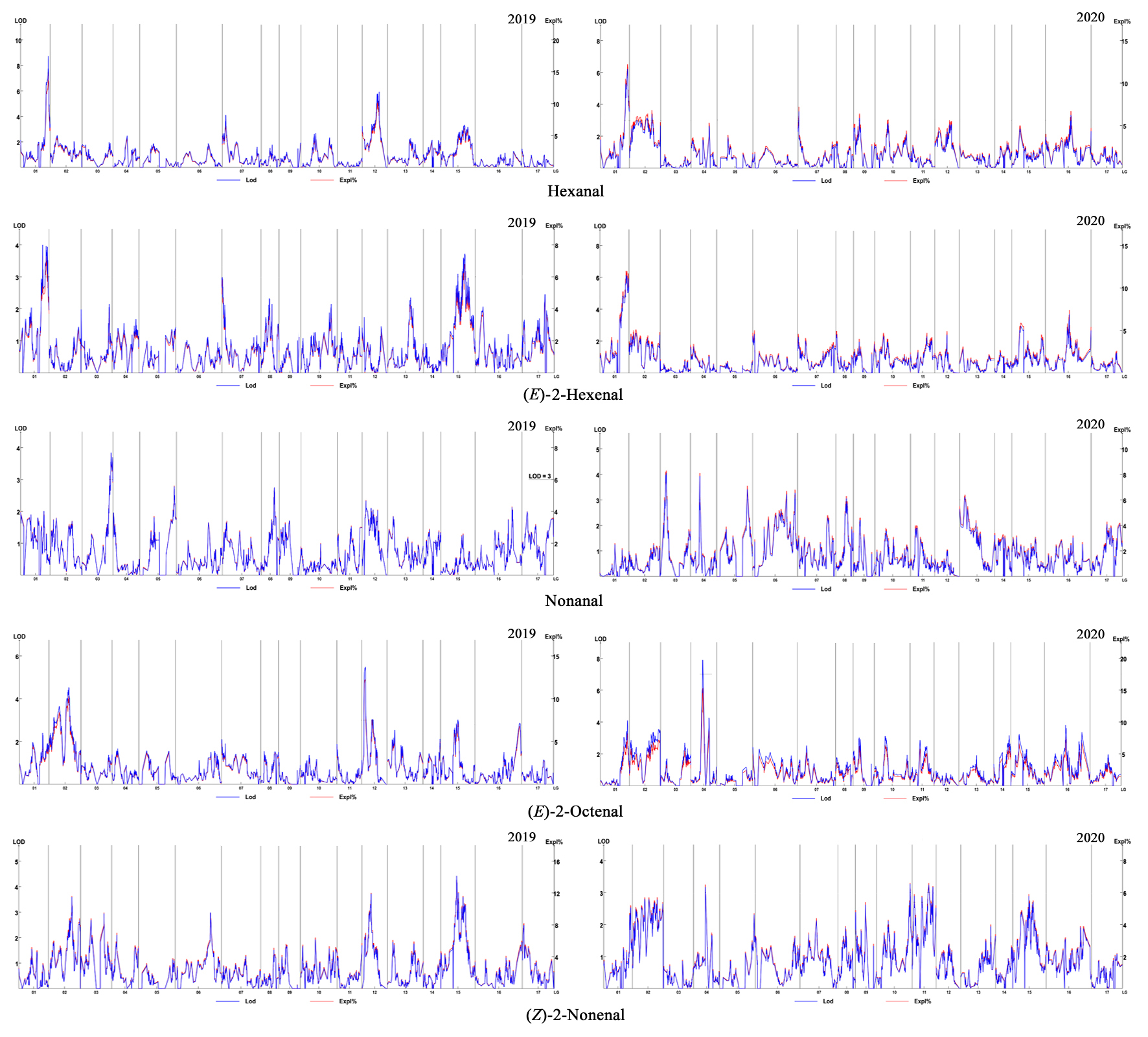


Figure S4 QTL mapping of five aldehyde compounds in 2019 and 2020


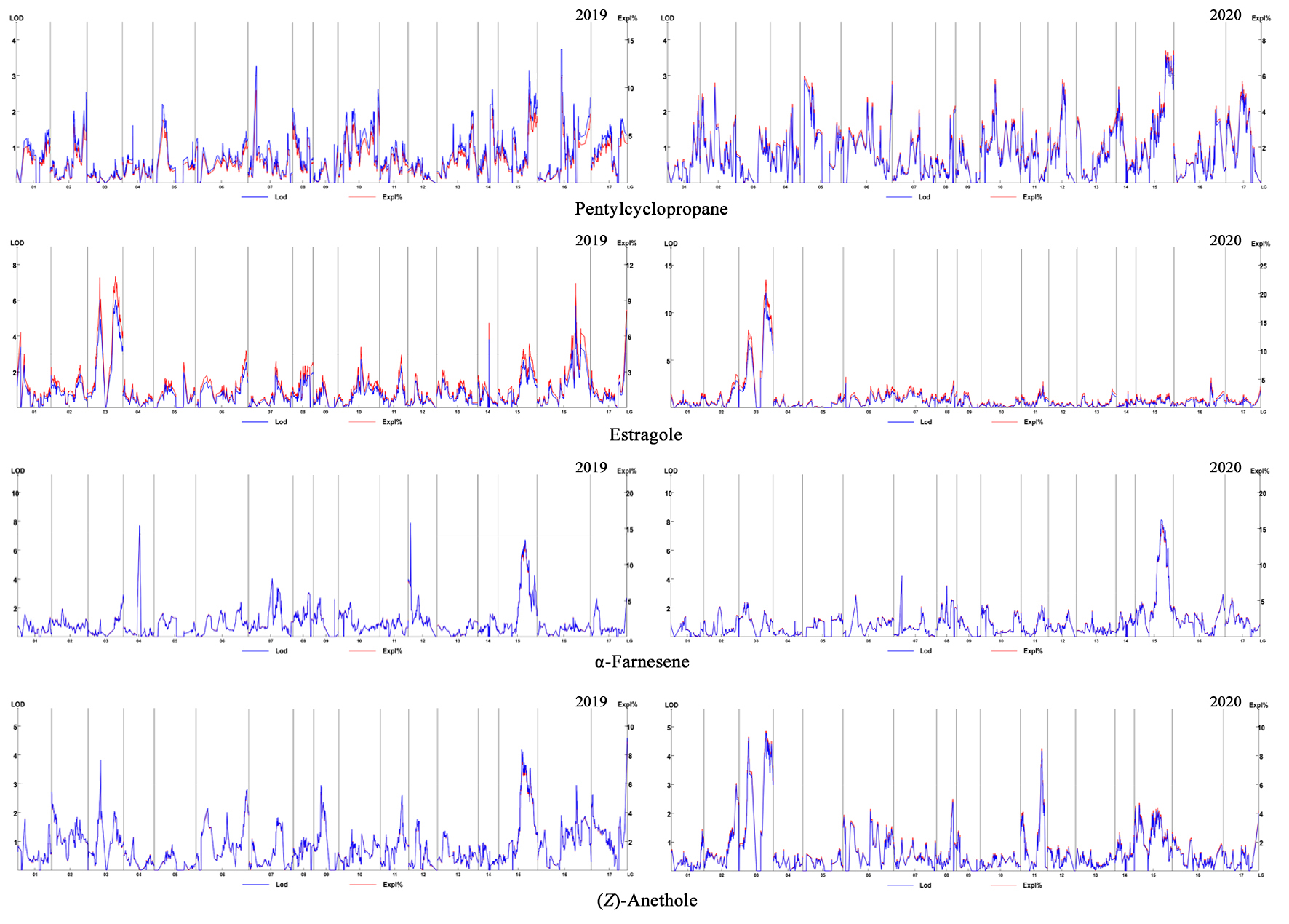


Figure S5 QTL mapping of four other compounds in 2019 and 2020

Table S1 The primers used for qRT-PCR and vector construction

| Gene | Forward primer (5'-3') | Reverse primer (5'-3') |
| --- | --- | --- |
| qRT-PCR |  |  |
| *MdAAT6* | CGAAAACAGATTCCACCC | CTCCAAAGCATATCCCAG |
| Cloning |  |  |
| *MdAAT6* | ATGATGCCATCCTCAGTACTTC | TTACATCATTGACATGATCCTA |
| pCAMBIA2300 |  |  |
| *MdAAT6* | acgagctcggtaccATGATGCCATCCTCAGTACTTC | catggtgtcgactctagaTTACATCATTGACATGATCCTA |
| TRV2 |  |  |
| *MdAAT6* | gtgagtaaggttaccgaattcCTACCATGAATGAAGAATACTT | gagacgcgtgagctcggtaccTTACATCATTGACATGATCCTA |

Table S2 The separation ratio and Chi-square test of segregated volatile compounds in F_1_ progeny fruits of ‘Fuji’ × ‘Cripps Pink’ population

| Volatile compounds | F1 progeny (2019) | | | | F1 progeny (2020) | | | |
| --- | --- | --- | --- | --- | --- | --- | --- | --- |
|  | Detected | Not-detected | TSR^a^ | *χ*^2^ test^b^ | Detected | Not-detected | TSR^a^ | *χ*^2^ test^b^ |
| Propyl acetate | 112 | 134 | 1:1 | 1.97 | 90 | 133 | 1:1 | 8.29 |
| Ethyl butanoate | 128 | 118 | 1:1 | 0.41 | 82 | 141 | 1:1 | 15.61 |
| Propyl propionate | 71 | 175 | 1:3 | 1.96 | 40 | 183 | 1:3 | 5.93 |
| Butyl acetate | 64 | 182 | 1:3 | 0.14 | 50 | 173 | 1:3 | 0.79 |
| 2-Methylbutyl acetate | 17 | 229 | 1:15 | 0.18 | 16 | 207 | 1:15 | 0.33 |
| Amyl acetate | 77 | 169 | 1:3 | 5.21 | 44 | 179 | 1:3 | 3.30 |
| Butyl butanoate | 41 | 205 | 1:3 | 9.11 | 15 | 208 | 1:3 | 13.49 |
| Butyl 2-methylbutanoate | 73 | 173 | 1:1 | 23.48 | 115 | 108 | 1:1 | 0.22 |
| Propyl hexanoate | 113 | 133 | 1:1 | 1.63 | 80 | 143 | 1:1 | 17.80 |
| Hexyl propanoate | 80 | 166 | 1:3 | 7.42 | 53 | 170 | 1:3 | 0.18 |
| Butyl hexanoate | 119 | 127 | 1:1 | 0.26 | 105 | 118 | 1:1 | 0.76 |
| 1-Butanol | 20 | 226 | 1:15 | 1.48 | 21 | 202 | 1:15 | 3.82 |
| Decanal | 35 | 211 | 1:15 | 26.72 | 7 | 216 | 1:15 | 3.68 |
| 1-Octen-3-one | 68 | 178 | 1:3 | 0.92 | 31 | 192 | 1:3 | 14.65 |

^a^ theoretical separation ratio. ^b^ Chi-square test ($\chi_{0.05,1}^{2}$=3.84)
